# Development and Validation of a Bioanalytical UHPLC-MS/MS Method Applied to Murine Liver Tissue for the Determination of Indocyanine Green Loaded in H-Ferritin Nanoparticles

**DOI:** 10.3389/fchem.2021.784123

**Published:** 2022-01-03

**Authors:** Cristina Sottani, Elena Grignani, Danilo Cottica, Serena Mazzucchelli, Marta Sevieri, Arianna Chesi, Fabio Corsi, Sarah Galfrè, Francesco Saverio Robustelli della Cuna, Enrica Calleri

**Affiliations:** ^1^ Environmental Research Center, Istituti Clinici Scientifici Maugeri IRCCS, Pavia, Italy; ^2^ Nanomedicine Laboratory, Department of Biomedical and Clinical Sciences “Luigi Sacco”, Milano University, Milan, Italy; ^3^ Breast Unit, Istituti Clinici Scientifici Maugeri IRCCS, Pavia, Italy; ^4^ Department of Drug Sciences, University of Pavia, Pavia, Italy

**Keywords:** indocynine green, liver tissue, UHPLC-MS/MS, FDA validation, biodistribution study

## Abstract

Indocyanine green (ICG) is one of the most commonly used fluorophores in near-infrared fluorescence-guided techniques. However, the molecule is prone to form aggregates in saline solution with a limited photostability and a moderate fluorescence yield. ICG was thus formulated using protein-based nanoparticles of H-ferritin (HFn) in order to generate a new nanostructure, HFn-ICG. In this study, an ultrahigh performance liquid chromatography-tandem mass spectrometry (UHPLC-MS/MS) system was employed to develop and validate the quantitative analysis of ICG in liver tissue samples from HFn-ICG-treated mice. To precipitate HFn, cold acetone in acidic solution at pH 5.0 was used. The processed liver samples were injected into the UHPLC-MS/MS system for analysis using the positive electrospray ionization mode. Chromatographic separation was achieved on a Waters Acquity UPLC^®^ HSS T3 Column (1.8 μm, 2.1 × 100 mm) with 0.1% formic acid and acetonitrile as the mobile phase with gradient elution. The selected reaction monitoring transitions of  m/z 753 →m/z 330 and m/z 827 →m/z 330 were applied for ICG and IR-820 (the internal standard, IS), respectively. The method was selective and linear over a concentration range of 50–1,500 ng/ml. The method was validated for sensitivity, accuracy, precision, extraction recovery, matrix effect, and stability in liver tissue homogenates. ICG extraction recoveries ranged between 85 and 108%. The intra- and inter-day precisions were less than 6.28%. The method was applied to a bio-distribution study to compare the amount of ICG levels from mice treated with HFn-ICG and free ICG. The analyses of the homogenate samples from the two types of treatment showed that the concentration levels of ICG is approximately six-fold higher than those of free ICG (1,411 ± 7.62 ng/ml vs. 235 ± 26.0 ng/ml) at 2 h post injection.

## Introduction

**FIGURE 1:**
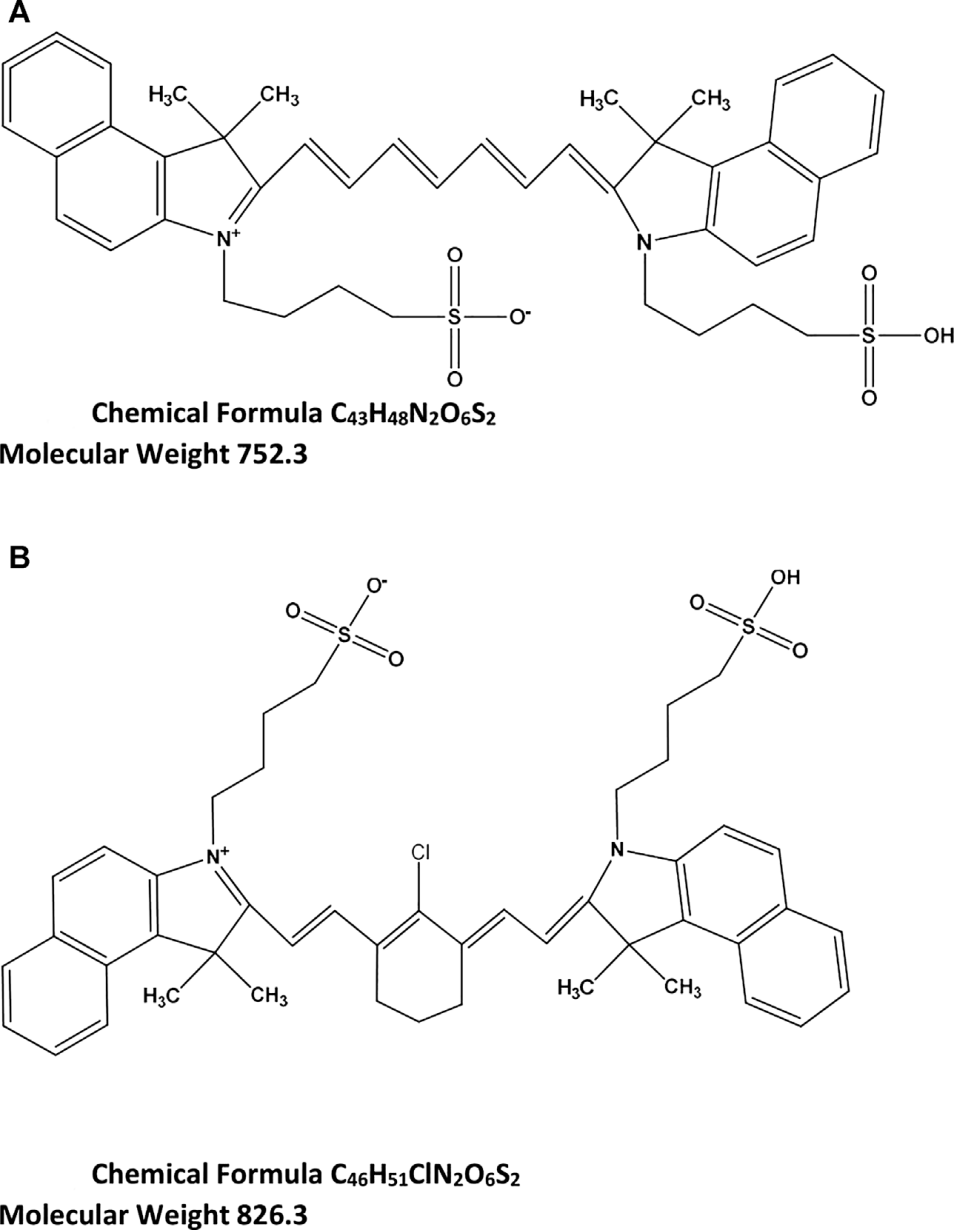
Chemical structure and formula of ICG **(A)** and IR-820 (IS) **(B)**.

Indocyanine green (ICG) (Figure 1) is a fluorescent dye which belongs to the family of cyanine dyes. ICG is an amphiphilic molecule, and this characteristic allows its dissolution in both aqueous and lipophilic solvents (Desmettre et al., 2000). Over the past decade, the molecule has become decisive as an *in vivo* imaging agent for different clinical applications (Reinhart et al., 2016; Ferrucci et al., 2018; Shen et al., 2018; Spinoglio et al., 2018; Sevieri et al., 2020). Moreover, as there is a growing interest in clinical imaging for diagnosis, staging, and therapy planning, this fluorescent dye has been investigated to develop a fluorescent probe with sufficient sensitivity in complex environments at deep-tissue penetration (Dang et al., 2019)**.** The availability of an FDA-approved agent combined with a highly sensitive second near-infrared window fluorophore has promoted studies for detection and treatment of various cancers (Bhavane et al., 2018; Wang et al., 2018; Egloff-Juras et al., 2019)**.**

However, ICG suffers from a high instability as the chemical degradation of this dye depends on the nature of solvents, concentration, temperature, and light exposure. In addition, the molecule tends to form aggregates in high concentrations (Desmettre et al., 2000). In order to increase its chemical stabilization, the amphiphilic nature of ICG has been used to generate nanostructures. In a recent study, ICG was loaded into nanocages of H-ferritin (HFn), a biocompatible protein, with the aim of studying a new nanotracer (HFn-ICG) in comparison to free ICG (Sitia et al., 2020). The accumulation of HFn-ICG in different organs of a breast cancer murine model was determined by using IVIS^®^ Lumina II *in-vivo* Imaging System (Sevieri et al., 2021)**.** For fluorescence pharmacokinetic rates of ICG in mouse liver, within the *in vivo* imaging techniques, diffusive fluorescence tomography has been also used (Zhang et al., 2018).

High performance liquid chromatography (HPLC) with ultraviolet, fluorescence, and tandem mass spectrometry (HPLC-MS/MS) have been used for determining the levels of free ICG in animal plasma and bile samples (Chen et al., 2015; Chen et al., 2008).

However, as ICG fluorescence varies, because of the dye concentration (i.e., the fluorescence quenches above 0.5 μg/ml), the clinical results are affected by incorrectly low fluorescence responses (Desmettre et al., 2000; Mindt et al., 2018). To measure ICG in both fluids and tissues, liquid chromatography tandem mass spectrometry (LC-MS/MS) technique has become the method of choice due to the high selectivity of mass spectrometric detector.

To our best knowledge, it is the first time that ultrahigh performance liquid chromatography tandem mass spectrometry (UHPLC-MS/MS) has been used to quantify HFn-ICG in mouse liver. More precisely, in our study, to analyze ICG encapsulated in the cavity of HFn, a simple and rapid sample clean-up procedure has been developed at the isoelectric point of HFn. Therefore, the accuracy of measuring ICG was achieved by first removing the protein-based interferences commonly present in biological samples. Moreover, the UHPLC-MS/MS assay used throughout the study was effective in the detection of ICG because not subject to impurities caused by a degradation phenomenon occurring in aqueous solution and followed by a rapid dimerization of ICG (Mindt et al., 2018).

The aim of the present study was to determine ICG levels in liver samples from mice treated with free ICG and ICG loaded in nanocages of HFn. The determination of ICG levels in tissues is essential for studying the tissue distribution of this compound.

In the present study, an accurate, specific, and reliable method with UHPLC-MS/MS was employed to quantify HFn-ICG using IR-820 as an internal standard (IS) Figure 1.

The developed procedure was also validated and applied to a bio-distribution study in liver homogenates from HFn-ICG-treated mice.

## Materials and Methods

### Chemicals and Reagents

Indocyanine green (90%, reference substance) and IR-820 dye (80%) powders were obtained from Ultra Scientific Analytical Solutions s.r.l. (Bologna, Italy). H-ferritin nanocages were produced as recombinant protein in *E. coli* as previously described (Bellini et al., 2014). For chromatography, acetonitrile (ACN) (LC-MS-grade), formic acid, and ammonium formate (LC-MS-grade) were purchased from Merck House, Poole, United Kingdom; acetone (99.9%) and hydrochloric acid (37%) were from Merck KGa A64271 Darmstadt, Germany; and water was deionized and purified on a Milli-Q system (Millipore, Marlborough, MA, United States).

### UHPLC-MS/MS Conditions

ICG quantification was performed using the UHPLC-MS/MS system consisting of an Agilent 1,290 Infinity Binary Pump, a 1,290 Infinity Sampler, a 1,290 Infinity Thermostat, and a 1,290 Infinity Thermostatted Column Compartment connected to an Agilent 6,460 triple quadrupole mass spectrometer (Agilent Technologies, Lexington, CA, United States). Mass Hunter workstation was used for data acquisition and analysis (Version 10.1 2006–2020). The instrument was operated in electrospray ionization, positive mode (ESI+) using Agilent Jet Stream. Ion source parameters were as follows: 11 ml/min, 300°C vaporizer temperature, 300 °C sheath gas, 600 V nozzle voltage, and 3000 V capillary voltage. Nitrogen was used as the nebulizer gas set at 45 psi with a flow rate of 5 L/min. ICG was quantitated at the selected reaction monitoring (SRM) transition of *m/z* 753 → *m/z* 330, and IR-820 was monitored at m/z 827 → m/z 330. The buffer was prepared by adding 1 ml 0.1% formic acid to 10 mM ammonium formate solution, pH 3.5. Several attempts were made to increase the ICG *y-axis* intensity by varying the ratio between the organic solvent and the aqueous solution (data not shown). Mobile phase A consisted of ACN:buffer, 20:80 (v/v), and mobile phase B ACN:buffer, 80:20 (v/v). ICG and IR-820A were separated using a Waters Acquity UPLC HSS T3 column (1.8 µm, 2.1 mm × 100 mm, Waters, Milford, MA, United States) maintained at 40°C with a flow rate of 0.3 ml/min.

The gradient programming started with mobile phase B 50% that was followed by a linear increase to 100% in 2.5 min, and maintained until 4 min. The system was allowed to return to initial conditions in 2 min. The total run time was 6 min.

### Sample Collection and ICG Encapsulation

The ICG was nano-formulated exploiting the ability of HFn nanocages to disassemble and reassemble its quaternary structure in response to changes in pH as previously reported (Sitia et al., 2020). A mixture of HFn (0.5 mg/ml) dissolved in 0.15 M NaCl was brought to pH 2.0 adding 0.5 M HCl. HFn was incubated at pH 2.0 for 15 min at room temperature by shaking at 100 rpm (OS-20 orbital shaker, BioSan, Italy) to disassemble the protein cage. Then, the pH was brought back to neutrality (pH 7.5) with the addition of 0.1 M NaOH. In the meantime, ICG powder Verdye (25 mg; Diagnostic Green GmbH, Aschheim-Dornach, Germany) was solubilized in bidistilled deionized water (5 ml; 5 mg/ml) and added to the HFn solution at a final dye concentration of 1.0 mg/ml. The mixture was incubated for 2 h at room temperature (RT) and shaking at 180 rpm to allow complete refolding of the HFn quaternary structure.

The obtained HFn−ICG nanoparticles were then concentrated by using Amicon Ultra-4 centrifugal filter devices (Merck S.p.a., Milan, Italy), and the non-encapsulated ICG has been removed by gel filtration using a Zeba Spin Desalting column (Thermo Fisher Scientific, Monza, Italy).

### Tissue Homogenization

Liver tissues from treated and untreated mice were explanted, accurately weighed, and homogenized in water (10% w/v) with potter homogenizer (Glas-Col homogenizer, IKA, China). Tissues were kept in the ice-bath during the process and stored at −0°C until use.

### Preparation of Standards and Quality Control Samples

Accurately weighed ICG powder was dissolved in methanol to prepare the stock standard solution at the concentration of 1 mg/ml and stored at −80°C in dark conditions. The IS IR-820 stock solution was prepared in methanol as well (1 mg/ml). Stock solutions in methanol were brought to 4°C to prepare the working standard solutions in methanol at the concentrations of 0.4, 0.8, 1.2, 3.2, 8.0, and 12.0 μg/ml for the calibration curve and 1.0, 2.0, 4.0, and 10 μg/ml for the quality control (QC) samples. Similarly, IS was prepared in methanol at the concentration of 4.0 μg/ml. Subsequently, each standard sample was prepared by adding 50 μl of each working standard solution and 50 μl of IS to a polypropylene tube. The aliquots were brought to dryness, and then 400 μl of blank liver tissue homogenate was added. A six-point calibration curve (50, 100, 150, 400, 1,000, and 1,500 ng/ml) with four QC levels at the concentrations of 125, 250, 500, and 1,250 ng/ml was obtained. The concentration level of IR-820 was 500 ng/ml.

Extraction of ICG and IS was performed using a cold protein precipitation (PP) solution according to a procedure previously described (Sottani et al., 2020). To each standard sample, an 8-fold volume (3,200 μl) of 1 mM PP solution was added. PP solution was prepared at pH 5.0 by addition of 10 μl 37% HCl to 120 ml of cold acetone. PP solution was kept at −80°C before use. For protein precipitation, the samples were vortexed for 1 min and centrifuged (14,000 rpm for 15 min, 4°C) to separate the supernatant from the pellet. For each sample, the supernatant was filtered with a 0.45 μm, 25 mm PTFE syringe filter and then dried under a gentle stream of N_2_. The dry residues were reconstituted with 100 μl of methanol, and 5 μl was injected in the UHPLC-MS/MS system.

### Assay Validation

The UHPLC-MS/MS method was validated according to Food and Drug Administration (FDA) Guidance on Bioanalytical Method Validation (Food and Drug Administration, 2018). The liver tissue homogenates from six animals were spiked with the IS only and with both ICG and IS. The selectivity of the assay was assessed comparing chromatograms of blank liver tissue to blank liver tissue spiked with the analyte.

### Linearity and Sensitivity

The linearity of the assay was evaluated using the standard samples prepared with ICG in homogenate of murine liver tissue over the concentration range of 50–1,500 ng/ml. Calibration curves were constructed by plotting the concentrations of ICG on the *x*-axis vs. the chromatographic peak area ratios of ICG to IS on the *y*-axis. Using the y = mx + b equation, the *y*-intercept (b), slope (m), and the coefficient of determination (R^2^) were calculated. The calibration curves were weighted using the weighting factor of 1/x. R^2^ ± SD values were used to evaluate the linearity of the assay using the criteria of ≥0.990.

The lower limit of quantification (LLOQ) was determined when the accuracy was between 80 and 120% and the precision ≤ 20%. The signal-to-noise ratio criterion of 10:1 was used to assess the LLOQ value.

### Accuracy and Precision

The accuracy and precision of the method were assessed by intra- and inter-day validation over three non-consecutive days. The intraday accuracy and precision were evaluated by processing QC samples in six replicates (*n* = 6) at four concentration levels. The concentration of the QC samples was calculated vs. the daily calibration curves. The inter-day accuracy and precision were determined by analyzing QC samples in 18 replicates of each concentration. Accuracy was determined as the ratio between the back-calculated concentration and the nominal value and expressed as a percentage. For precision, the mean ratio of ICG peak area/IS peak area was used. The coefficient of variation (CV%) was employed as a measure of precision.

### Recovery and Matrix Effect

The percentage extraction recovery (RE) was calculated at the lower level of quantification for ICG and at the liver tissue concentrations of the four QC samples (125, 250, 500, and 1,250 ng/ml) that were prepared as six replicates. The mean integration ratio (area of the analyte/area of IS) for ICG and IS spiked before PP extraction (Set C) was compared to that of ICG and IS spiked in the matrix of extracted blank homogenate of liver tissue (Set B). The mean peak area of IS prepared at the concentration of 500 ng/ml was used to assess its recovery from liver samples.

Matrix effect (ME) was assessed by comparing the mean ratio of the peak areas (ICG/IS) spiked in blank liver tissue after PP procedure to that of ICG standard solutions prepared in methanol (Set A). The potential for the relative matrix effect was also evaluated using six independent sources of extracted blank homogenates of murine liver tissues. ICG was therefore spiked at the QC levels in six blank liver tissue samples. The mean integration ratio of ICG for the four QC samples of Set B was compared to that of the QC standard solutions at the same concentrations (Matuszewski et al., 2003).

### Stability

The stability of ICG in homogenate liver samples was assessed by analyzing QC samples at two concentration levels (125 and 500 ng/ml) during storage and handling. Bench-top stability was determined at room temperature after 8 h of the handling of ICG samples. Stability in the autosampler was also assessed at RT by reanalyzing the processed QC samples 48 h after the first injection. Freeze-thaw stability was studied by analyzing QC samples that were frozen overnight, at normal storage temperature (−80°C), and thawed at 4°C. When completely thawed, the samples were frozen again at the same temperature for 24 h and thawed. This freeze-thaw cycle was repeated two more times. After the third cycle (4 days), the samples were analyzed. To check freeze–thaw stability, an aliquot of each QC sample concentration was freshly prepared, processed, and analyzed. The analyte was considered stable at each concentration when the differences between the freshly prepared samples and the stability testing samples did not vary more than ±15% from the nominal concentrations.

### Application to Bio-Distribution Studies in Liver Homogenates

The animals were managed according to procedures approved by the Italian Ministry of Health (Protocol Number 611/2019-PR, August 6, 2019). All procedures involving animals and their health were conducted in accordance with the 3R principles to minimize the number of mice used and their collateral suffering. The animals were housed in specific pathogen-free conditions and were kept in cages with free access to water and food. In this study, a total of 32 seven-week-old female BALB/c mice (average weight of 20 g) were recruited and randomly divided in four groups (blank, 2, 6, and 24 h). For the method development, six animals were used as blank controls. Animals were intravenously injected in the tail vein with ICG or HFn−ICG at a dose of 3.8 mg/kg. Thus, the number of murine tissue samples was as follows: *n* = 8 at 0 and *n* = 8 at 2 h, *n* = 8 at 6 h, and *n* = 8 at 24 h. For each group, four animals were treated with free ICG and four animals treated with ICG loaded in HFn nanoparticles. Subsequently, the mice were sacrificed by cervical dislocation and the collected liver samples were stored at −80°C until use. Two way ANOVA was used, where time and the two types of treatment, and the interaction between them, have been applied using the R software tool (www.r-project.org). This study was part of a more complete and thorough investigation about ICG and HFn-ICG distribution in different murine tissues. The outcomes of this research will be reported in detail in another specific paper.

## Results and Discussion

### Method Development and Validation

#### Mass Spectrometry

**FIGURE 2:**
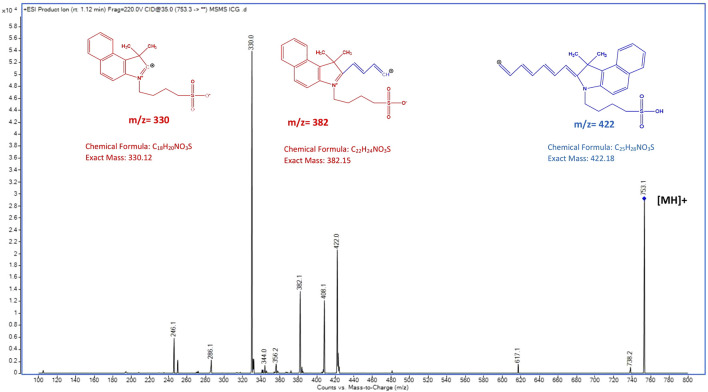
Product ion mass spectrum of ICG obtained with electrospray ionization, positive mode.

The chemical structures of ICG and IR-820 are shown as ionized forms in Figure 1. With ESI+, ICG showed a protonated molecular ion [MH]^+^at m/z 753.2, and the IS at m/z 827 (not shown in Figure 2). In Figure 1, the molecular structure of ICG is presented as a combination of red and blue colours. Figure 2 shows the product ion mass spectrum of ICG where red represents the main fragment ion of ICG at m/z 330, and blue at m/z 422. Other fragment ions are m/z 382 and m/z 408 (Figure 2).

In the product ion spectra of ICG reported in Figure 2, other fragment ions are m/z 382 and m/z 408. As the molecular structure of ICG is symmetric and is composed of two polycyclic parts (benzoindotricarbocyanin) linked by an alkene chain, the fragment ions such as those at m/z 382 and m/z 408 are observed indicating a fragmentation at the unsaturated chain of ICG.

Thereafter, the selected reaction monitoring (SRM) for quantitative and qualitative analyses was performed using the following transitions: m/z 753→330, m/z 753→422 for ICG, and m/z 827→330 for IS.

### Selectivity, Linearity, and LLOQ

**FIGURE 3:**
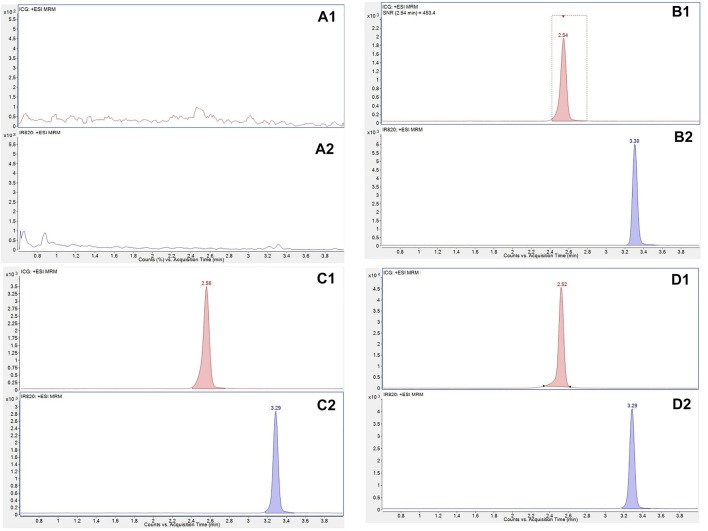
Representative SRM chromatograms of ICG **(A1)** and IR-820 **(A2)** in blank murineliver tissue; in murine liver tissues samples spiked with (50.0 ng/ml) showing a reaction time (RT) 2.6 min **(B1)**; and with IR-820 (500 ng/ml) showing a RT 3.3 min **(B2)**. Representative SRM chromatograms of tissue samples obtained 2 h post injection from mice treated with free ICG **(C1)** and HFn-ICG **(D1)**. For the two types of treatment, the SRM chromatograms for IR-820 are presented in **(C2)** and **(D2)**.

Figure 3 shows the SRM channels of ICG (A1) and IR-820 (A2) in blank murine liver tissue. No interferences were found at the retention times of ICG and IR-820. Figure 3B1 represents a murine tissue sample spiked with ICG (50 ng/ml) showing a retention time (Rt) 2.6 min, and (B2) spiked with IR-820 (500 ng/ml) showing a Rt 3.3 min.

The PP procedure carried out at the isoelectric point of HFn (pH 5.0) with cold acetone was effective in the selective recovery of an amphiphilic molecule like ICG from homogenate samples of liver tissue.

The analytical conditions used to extract the target molecule from omopolymers of H-ferritin were therefore considered adequate as symmetrical and well baseline-separated peaks of ICG and the IS were obtained.

In addition, in order to show the different concentration levels of ICG in murine liver tissue, typical SRM chromatograms of ICG from mice treated with free ICG and HFn-ICG are presented in Figures 3C1,D1. In this figure, the concentrations of the fluorescent dye at 2 h post injection are reported. The level of ICG delivered by nanoparticles was higher (1,411 ng/ml) (C1) than that of free ICG (206 ng/ml) (D1). The SRM signals of IR-820 obtained with the two types of dosing are presented in Figures 3C2,D2.

The concentration levels detected from mice treated with free ICG and HFn-ICG decreased over time and detectable levels of ICG were determined up to 24 h. The lowest concentration value of ICG at 24 h was 56.7 ng/ml. For this reason, the calibration curves (integration ratio values vs. concentration values) for ICG in murine liver tissue were studied over a range of 50–1,500 ng/ml. The calibration equation is represented by *y* = (m ± SD) x − (b ± SD) where *y* is the relative response (peak area ratios ICG-to-IS) and x is ICG concentration in murine tissue. R^2^ is the coefficient of determination ± SD. The calibration equations prepared in triplicate over the 3 days of the validation process showed to be linear under the developed analytical conditions using the weighted (1/x) least squares regression analysis. The mean calibration equation for ICG was *y* = (0.00741 ± 0.000282) x − (0.0918 ± 0.0585), with R^2^ = 0.996 ± 0.000245. As shown in Figure 3 (panel B1), the high signal-to-noise ratio (≥ 400) of ICG obtained by injecting the lowest calibration standard spiked in blank liver tissue samples allowed a good sensitivity of the UHPLC-MS/MS assay with the LLOQ at 50 ng/ml which is around the expected concentrations of HFn-ICG in the matrix. The LLOQ response satisfied the acceptance criteria as the inter-day accuracy was 101% and the precision was 4.32%. Intra-day accuracy and precision are reported in Table 1.

**TABLE 1:** Accuracy and precision intra- and inter-day of ICG in murine tissue samples.

Nominal concentration (ng/ml)	Liver tissue					
	Accuracy (%)			Precision (CV, %)		
***Intra-day (n = 6)***						
	Day 1	Day 2	Day 3	Day 1	Day 2	Day 3
50.00	100	99.8	104	3.29	2.17	5.67
125.00	108	106	108	4.78	10.1	2.67
250.00	91.2	92.3	97.8	7.29	5.87	2.80
500.00	91.2	93.1	89.1	4.90	6.50	2.09
1,250.00	98.9	103	107	1.15	2.03	1.69
***Inter-day (n = 18)***						
50.00	101			4.32		
125.00	107			6.28		
250.00	93.8			5.80		
500.00	91.2			5.12		
1,250.00	103			3.99		

### Accuracy and Precision

QC samples were analyzed in six replicates over three different days to determine the intra-day accuracy and precision. Four QC levels of ICG in murine liver tissues were investigated. Table 1 summarizes the accuracy and precision for the obtained concentrations at the QC levels. Accuracy and precision for ICG determination in liver samples were within the acceptable limits recommended by the FDA bioanalytical method validation guideline. Hence, inter-day accuracy ranged from 91.2 to 107% and the intra-day precision was included between 3.99 and 6.28% for ICG detected in liver tissue samples.

### Recovery and Matrix Effect

The results of recovery and matrix effect are presented in Table 2. The recovery of ICG from homogenate liver samples was evaluated by comparing the peak area ratio of ICG/IS QC samples (Set C) with that of blanks spiked with the analyte after PP procedure at the same concentrations. The extraction recovery values were similar at the LLOQ level and over the concentrations of the four quality control samples (50–1,250 ng/ml). They ranged from 84.8 to 108% with a precision that met the requirements of FDA guideline (Table 2). For the absolute matrix effect, the mean integration ratio values of ICG QC samples extracted (Set B) were compared with those of the standard solutions (Set A) at the same concentrations. The absolute matrix effect in murine liver tissue for ICG ranged from 77.0 to 103% (Table 2). There was no significant effect of the matrix for the analysis of ICG in liver tissue samples using the developed UHPLC-MS/MS method.

**TABLE 2:** Extraction recovery and matrix effect of ICG in murine liver tissue samples.

Concentration	Set B	Set C	Recovery (%)	Precision (CV%)
(ng/ml)	Mean int. ratio ± SD	Mean int. ratio ± SD		
ICG				
50.0	0.280 ± 0.020	0.306 ± 0.036	107	11.8
125	0.852 ± 0.070	0.920 ± 0.097	108	10.6
250	1.90 ± 0.066	1.64 ± 0.074	87.0	4.47
500	3.83 ± 0.253	3.25 ± 0.255	84.8	7.85
1,250	9.66 ± 0.716	9.52 ± 0.254	98.6	2.67
IR-820 (ISTD)	Mean area	Mean area		
500	32,976 ± 5,088	31,396 ± 4,010	95.2	12.8
**Concentration**	**Set A**	**Set B**	**Matrix effect (ME%)**	**Precision (CV%)**
**(ng/ml)**	**Mean int. ratio ± SD**	**Mean int. ratio ± SD**		
ICG				
125	1.11 ± 0.140	0.852 ± 0.070	77.0	8.36
250	2.07 ± 0.270	1.90 ± 0.066	91.7	1.82
500	3.98 ± 0.572	3.83 ± 0.253	96.2	6.61
1,250	9.37 ± 1.35	9.66 ± 0.716	103	7.42

### Stability

The results of stability are presented in Table 3. At short-term storage conditions (48 h, at −80°C), stock solutions of ICG showed the stability of 80.2 and 78.2% compared to fresh-prepared solutions prepared at the concentration of 50.0 ng/ml and 1,250 ng/ml. It is important to underline that stock solutions of ICG in methanol showed concentration levels less than 60% up till 48 h at −80°C if compared to those freshly prepared. Therefore, they have to be prepared freshly every day and stored in dark conditions. The working standard solutions in methanol are stable for up to 72 h at −20°C within 85.1% at the lowest levels of the QC samples. The working standard solutions have to be freshly prepared every 3 days over the development of the validation procedure. The stability of ICG was investigated also in liver samples. For bench top stability, the concentration values of ICG decreased by 15% during the handling of ICG homogenates of liver tissues when the samples were maintained in lab at RT (approximately 6 h). The stability of extracted samples stored in autosampler was measured up to 48 h (room temperature) and showed approximately 11% drop in the ICG levels.

**TABLE 3:** Stability of ICG in standard solutions and in murine liver tissue samples.

Storage condition	Concentration (ng/ml)	Stability (%)
Stock solution (48 h, −80°C)	50.0	80.2
	1,250	78.2
Working standard (72 h, −20°C)	50.0	85.1
	1,250	91.4
Storage condition	Concentration (ng/ml)	Liver tissue stability (%)
Bench-top (6 h, 25°C)	50.0	85.1
	1,250	87.2
Processed sample stability (48 h, 25°C)	50.0	90.1
	1,250	88.1
Freeze-thaw (3 cycles)	50.0	81.1
	1,250	85.3

After three freeze and thaw cycles, changes in responses of ICG spiked in the liver samples were in acceptable ranges (Table 3).

### Applicability to Bio-Distribution Studies

The validated analytical method was used to determine the concentrations of ICG formulated as HFn-ICG. In this study, we compared the ICG concentrations obtained from the liver tissue samples of four mice treated with HFn-ICG and free ICG at three different time points. For each point (2, 6, 24 h), four animals were treated with HFn-ICG and four animals with free ICG. The average results were obtained for the two different types of treatments. The maximum concentration of ICG was obtained at 2 h post injection showing that the concentrations of ICG in liver from mice treated with HFn-ICG were approximately six-fold higher than those from mice treated with free ICG. As an example, the concentrations of ICG observed at 2 h are presented in Figures 3C1,D1. Indeed, the results indicated a significant difference in the concentration values between ICG quantified by analyzing liver samples from mice treated with HFn-ICG and mice treated with free ICG (1,411 ± 7.62 ng/ml vs. 235 ± 26.0 ng/ml). The significant difference became negligible at 6 h. The analyses of liver tissue samples from mice treated with HFn-ICG and free ICG showed that the mean concentration values were 89.6 ± 22.5 ng/ml and 71.8 ± 8.75 ng/ml, respectively. Furthermore, this study revealed that the concentrations of ICG slowly dropped to 68.8 ± 17.2 ng/ml at 24 h for mice, group 1, treated with HFn-ICG and to 56.7 ± 4.20 ng/ml at 24 h for mice of the same group treated with free ICG. The present method enabled the determination of HFn-ICG up to 24 h in liver samples of the studied murine model. The results are summarized in Table 4. Two way ANOVA where time (in hours) and the two types of treatment, and the interaction between them, have been used as the explanatory variables and concentration (ng/ml) as the response variable confirmed a statistically significant interaction between time and treatment in modulating concentration (*p* < 0.001), assuming a significance level corresponding to alpha = 0.05. Due to the small sample size and the limited number of observations, because the bio-distribution study is in its early stage, the results should be cautiously interpreted.

**TABLE 4:** HFn-ICG and free ICG concentrations in murine tissue samples.

Treatment	Time (h)		Treatment	Time (h)		*p* Value
HFn-ICG		Conc. (ng/ml ± SD)	ICG		Conc. (ng/ml ± SD)	
	2	1,411 ± 7.62		2	235 ± 26.0	*p* = <0.001
	6	89.6 ± 22.5		6	71.8 ± 8.75	
	24	68.8 ± 17.2		24	56.7 ± 4.20	

## Conclusion

In this study, an UHPLC-MS/MS bioanalytical method was successfully developed and validated for the determination of ICG levels in murine liver tissue. The sample preparation required PP procedure for ICG extraction. This method exhibited adequate selectivity, linearity, and sensitivity to obtain accurate and precise measurements of ICG in homogenate samples prepared from mice treated with free ICG and ICG loaded in protein-based nanoparticles. The UHPLC-MS/MS method can be applied in bio-distribution studies to investigate the delivery of this new formulated fluorescent dye (HFn-ICG).

## Data Availability

The original contributions presented in the study are included in the article/Supplementary Material. Further inquiries can be directed to the corresponding authors.
